# “I Invest by Following Lead Investors!” The Role of Lead Investors in Fundraising Performance of Equity Crowdfunding

**DOI:** 10.3389/fpsyg.2020.00632

**Published:** 2020-04-24

**Authors:** Tao Shen, Jiangshui Ma, Bin Zhang, Wen Huang, Fan Fan

**Affiliations:** ^1^School of Business Administration, Southwestern University of Finance and Economics, Chengdu, China; ^2^School of Foreign Languages for Business, Southwestern University of Finance and Economics, Chengdu, China; ^3^School of Finance, Southwestern University of Finance and Economics, Chengdu, China; ^4^Research Institute of Economics and Management, Southwestern University of Finance and Economics, Chengdu, China

**Keywords:** equity crowdfunding, lead investors, signaling theory, observational learning theory, fundraising performance

## Abstract

Psychological factors play a critical role in affecting investor decisions. This study explores how lead investors influence following investors psychologically, thus affecting fundraising performance of equity crowdfunding. We draw upon the signaling theory and observational learning theory to argue that following investors could be induced to invest in a project if they observe the proportion of funding by lead investors in the funding target to be high, that the lead investors have rich investment experience, and that the lead investors can offer help to the projects. To test our hypotheses, we analyze a sample of 215 projects from a Chinese equity crowdfunding platform. The results reveal that the proportion of lead investor investment in the funding target and their investment experience are positively related to fundraising performance. However, the help offered by lead investors toward the projects has no impact on funding performance. Theoretical and practical implications are discussed.

## Introduction

Crowdfunding has emerged as a viable approach for new ventures to obtain external funding and has grown rapidly (Bruton et al., 2015; Short et al., 2017; Block J. H. et al., 2018). The global crowdfunding volume was estimated in 2015 at $34 billion, an increase of more than 112% over the 2014 volume^1^. The World Bank believes that crowdfunding could account for over $300 billion in cumulative transactions by 2025 (Meyskens and Bird, 2015). By definition, crowdfunding refers to the efforts by entrepreneurial individuals and groups to fund their ventures by drawing on relatively small contributions from a large number of individuals using the Internet, without standard financial intermediaries (Mollick, 2014). Primarily, there are four forms of crowdfunding: donation-based, reward-based, debt-based, and equity-based crowdfunding (Bi et al., 2017). In donation-based crowdfunding, funding is raised for charitable purposes. In reward-based crowdfunding, funders receive a reward for their contribution to a project. Debt-based crowdfunding is the practice of lending money to individuals or businesses with the expectation of some monetary returns. In equity-based crowdfunding, entrepreneurs raise funding from individual investors by providing them equity.

Though crowdfunding has surged in popularity in the last decade and now accounts for billions of dollars annually, scholarly knowledge about crowdfunding remains limited (Short et al., 2017). There is even less scholarly understanding about equity crowdfunding (Kuppuswamy and Bayus, 2018), which is the focus of the current study. Equity crowdfunding denotes a form of financing in which entrepreneurs make an open call to sell a specified amount of equity or bond-like shares in a company on the Internet, hoping to attract a large group of investors (Ahlers et al., 2015). Studies have begun to investigate the factors that drive the campaign success of equity crowdfunding (e.g., Ahlers et al., 2015; Lukkarinen et al., 2016; Vismara, 2016; Block J. H. et al., 2018). Most of these studies use signaling theory (Spence, 1973) as the theoretical mechanism to understand the funding success of equity crowdfunding. Ahlers et al. (2015), for example, argued that venture quality (i.e., human capital, social capital, and intellectual capital) and uncertainty are critical signaling to investors, thus, influencing their funding decisions. In a similar vein, Vismara (2016) investigated the signaling role of equity retention and social capital, both of which were found to influence investors’ decision toward equity crowdfunding projects.

While the research applying the signaling theory in equity crowdfunding context has predominantly focused on signals of venture (Chan and Parhankangas, 2017), entrepreneurs (Ahlers et al., 2015), equity retention (Vismara, 2016), and so on, little is known about how signals of lead investors influence the funding decision of subsequent investors. This limits our understanding on how lead investors can be managed to increase the fundraising performance of equity crowdfunding projects. The term “lead investor” is relatively new in the crowdfunding context. In the four forms of crowdfunding, it only exists in equity crowdfunding platforms. Lead investors conduct due diligence on projects and take the initiative of investing in a project, which they believe to have potential. Other small investors can choose to follow lead investors to make subsequent investments on the same project. In this study, we propose that lead investors can affect other investors’ investment behavior, which, in turn, influences the fundraising performance of equity crowdfunding projects. On one hand, based on signaling theory (Spence, 1973), the characteristics and investment behavior of lead investors could signal the quality of projects, thus, improving the sense of psychological safety of other investors and inducing them to invest the same projects. On the other hand, people sometimes make decisions by merely looking at others’ behavior, a phenomenon known as observational learning (Bikhchandani et al., 1998). It is a normal psychological phenomenon that people observe others’ behavior before choosing their own actions. This gives them some references on whether to engage in the same behavior. For example, before buying something, many people prefer to observe if their friends have bought the same thing. If many of their friends have bought it, they may be more likely to buy it. In contrast, if few of their friends have bought it, they may hesitate to buy. In the context of equity crowdfunding, investors face high uncertainty at the beginning of crowdfunding campaigns. They may make their investment decisions by looking at the investment behavior and characteristics of lead investors, who are usually more professional than they are. The investment behavior and characteristics of lead investors are likely to give them more (or less) psychological safety when making the investment decision. Given the potential importance of the role of lead investors in the fundraising performance of equity crowdfunding, we are surprised that few studies have investigated how lead investors influence the equity crowdfunding performance.

**FIGURE 1:**
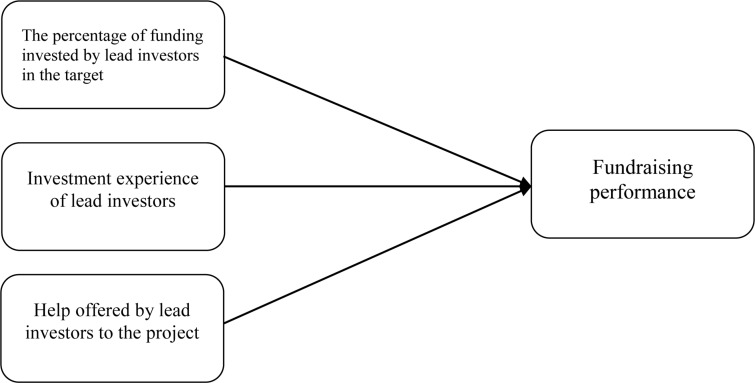
Conceptual model of the study.

To unfold the role of lead investors in equity crowdfunding, this study investigates the signaling role of lead investors on the investment decisions of subsequent investors and, ultimately, on the fundraising performance of equity crowdfunding. Specifically, we examine three variables of lead investors: the percentage of funding provided by lead investors out of the total crowdfunding goal, the investment experience of lead investors, and the help offered by lead investors to the crowdfunding project. Figure 1 presents the conceptual model of this study, which contributes to the literature in three substantive ways. First, we help to advance the knowledge about factors of equity crowdfunding performance by unraveling the role of lead investors. To the best of our knowledge, we are the first to explore the role of lead investors in the fundraising performance of equity crowdfunding. Second, we are also the first to apply observational learning theory into the equity crowdfunding context. Third, the findings of this study have important practical implications for both entrepreneurs who want to raise money through equity crowdfunding and equity crowdfunding platforms.

The rest of this paper is organized as follows. First, we provide a literature review of the research on crowdfunding and equity crowdfunding. We then provide the theories and hypotheses. Next, we present the data and methodology. After that, we present and discuss the results. Finally, we conclude the paper and discuss the limitations as well as future research directions.

## Literature Review

There is an increasing interest in the drivers of crowdfunding campaign success (Lagazio and Querci, 2018). In this stream of research, signaling theory has been widely used. In particular, scholars have mainly focused on the signals of project quality and individual (entrepreneur) quality when investigating the success factors of fundraising performance of crowdfunding (Kuppuswamy and Bayus, 2018). Mollick (2014), for instance, found that personal networks and underlying project quality are associated with the success of crowdfunding efforts. Zheng et al. (2014) demonstrated that an entrepreneur’s social network ties, obligations to fund other entrepreneurs, and the shared meaning of the crowdfunding project between the entrepreneur and the sponsors positively impact crowdfunding performance. Colombo et al. (2015) found that entrepreneurs’ internal social capital predicts the success of a crowdfunding campaign. Ahlers et al. (2015) found that project quality, characterized by retaining equity and providing more detailed information on risks, serves as effective signals that can influence funding performance.

Compared with the other forms of crowdfunding, equity crowdfunding has received limited attention regarding the drivers of fundraising performance. In fact, there is little research on equity crowdfunding, in general. In a review of crowdfunding research, there were only three studies on equity crowdfunding out of 42 studies (7%) on crowdfunding (Kuppuswamy and Bayus, 2018). One reason for this could be that equity crowdfunding is a relatively new concept that is subject to various regulatory issues, making it restricted until now in many countries. In the United States, for example, entrepreneurs were not allowed to obtain financing from “non-accredited” investors in exchange for equity shares through equity crowdfunding until October 2015 when Title III of Jumpstart Our Business Startups Act was passed. However, with more countries permitting equity crowdfunding, knowledge of equity crowdfunding as well as the factors of fundraising performance inequity crowdfunding is becoming necessary. Therefore, some scholars argue that equity crowdfunding is likely to be the subject of many research papers in finance and management in the coming years and encourage scholars to conduct more research on this topic (Vulkan et al., 2016).

Some studies have examined the factors of funding performance of equity crowdfunding (e.g., Ahlers et al., 2015; Lukkarinen et al., 2016; Vismara, 2016). Ahlers et al. (2015) used signaling theory to examine the impact of firms’ financial roadmaps, external and internal governance, and risk factors on fundraising success and found that human capital, amount of equity offered, and financial projections positively influence the success of equity crowdfunding campaigns. Vismara (2016) found that a larger percentage of equity offered by founders reduces the probability of equity crowdfunding campaign success, while a larger number of founders’ social connections increase the probability of equity crowdfunding campaign success. Lukkarinen et al. (2016) demonstrated that campaign success is related to pre-selected crowdfunding campaign characteristics and the utilization of private and public networks. Though these studies have enriched our understanding of the factors of equity crowdfunding campaign success, little is known about how lead investors influence the funding success of equity crowdfunding.

### Lead Investors in Equity Crowdfunding

The lead investor–follower investor model originally comes from AngelList, a leading equity crowdfunding platform in the United States. AngelList uses syndicates as an investment mode (Agrawal et al., 2016). Individual angel investors create a syndicate online profile, in which potential backers can view some basic information, like how many deals the angel investors expect to syndicate and how much they typically invest each year. Backers can apply to follow the syndicate lead. If accepted, the backer agrees to invest in the lead’s syndicated deals on the same terms as the lead and to pay the lead a carry (Agrawal et al., 2016).

In China, the lead investor–follower investor model has started to appear in equity crowdfunding platforms. One of the rationales for adopting this model is that the risk faced by small investors is reduced when following lead investors, who are usually more professional and can conduct due diligence on projects. The leader–follower model in Chinese equity crowdfunding is different from the syndicate model of AngelList. The following investors on Chinese equity crowdfunding platforms are not bound to follow the lead investors. They can make their decisions independently, and they can invest simultaneously in different projects led by different lead investors without paying a carry. Lead investors conduct due diligence on projects and serve as the first investor on a project that they believe is of good quality. Once a lead investor invests in a project, the website lists the information on lead investors, such as the lead investors’ comments on projects, their investment experience, how much they invested on a project, whether they can offer other help to the project, and so on. Based on signaling theory and observational learning theory, the follower investors could watch the investment behavior of lead investors and rely on their signals in addition to the signals from entrepreneurs and projects. Therefore, lead investors could, ultimately, influence the funding performance of equity crowdfunding projects.

## Theory and Hypotheses Development

The ability to signal quality to potential investors is crucial in gaining venture finance. Firms can use signals to decrease the information asymmetry between the firm and the investors (Beatty and Ritter, 1986). Signaling theory (Spence, 1973), therefore, becomes one of the main research frameworks for studying entrepreneurial finance (e.g., Carter and Manaster, 1990; Connelly et al., 2011; Migliorati and Vismara, 2014; Judge et al., 2015). The signaling theory originates from the works of Spence (1973). It suggests that the behavior of individuals or organizations, in the midst of information asymmetries, depends on how the senders send signals and how the decision makers interpret them (Connelly et al., 2015). In organizational research, the signals examined by researchers include the composition and structure of top management (Judge et al., 2015), corporate governance characteristics (Audretsch and Lehmann, 2013), firm’s social networks (Carter et al., 1998), and so on. Like the other forms of venture financing, equity crowdfunding also faces information asymmetries. Therefore, it is appropriate to apply the signaling theory in this context.

On equity crowdfunding platforms, entrepreneurs set a funding goal for their project and disclose information online to convince investors to support their projects. Investors rely heavily on the released information to make their funding decisions. However, compared with the traditional investors, such as angel investors and venture capitalists, crowdfunding investors are less capable of overcoming information asymmetry problems. They typically lack the capability to evaluate different investment opportunities and have limited opportunities to perform due diligence (Ahlers et al., 2015). According to the observational learning theory (Bikhchandani et al., 1998), consumers or investors’ buying or investing behavior is largely influenced by others’ behavior when the quality of a product or a project is not directly observable. For example, on-line consumers prefer to buy goods that are rated the highest. Therefore, when making investment decisions, small investors watch someone who is more professional and can perform due diligence. Based on this, we expect that the signals sent by lead investors may exert substantial influence on how potential investors perceive the projects, thus, affecting their investment decisions.

As earlier discussed, previous studies have investigated the signals of venture quality, project uncertainty, equity retention, entrepreneur’s social capital, and so on in influencing the success of equity crowdfunding (e.g., Ahlers et al., 2015; Vismara, 2016). However, the role of lead investors has been neglected. Note that the signals sent by entrepreneurs may not always be effective or trustworthy. In some cases, entrepreneurs tend to be over optimistic or have a natural incentive to exaggerate their prospects and the potential value of their firm (Cooper et al., 1988). Lead investors, however, have different interests than the entrepreneurs. They try, as much as they can, to obtain true information from the firms because they do not want to lose money. Therefore, investors in equity crowdfunding platforms may have strong incentives to make investment decisions by relying on the information of lead investors (Mohammadi and Shafi, 2018), who have the ability to master more information on equity crowdfunding projects and are neutral to some degree. We focus on three variables of lead investors: the percentage of funding provided by the lead investors out of the funding target, the investment experience of lead investors, and the help offered by lead investors toward the projects because they are notable variables, which may influence investment performance (Barry et al., 1990; Gompers, 1995; Busenitz et al., 2005; Seru et al., 2009).

On some Chinese equity crowdfunding platforms, the amount of capital collected by a project and the investment information of the lead investors are visible to potential investors. For instance, Dajiatou, a Chinese equity crowdfunding website, displays the information of lead investors on the top right of its project pages, so that visitors can view who have taken the lead to invest in a project and what percentage of capital out of the target has been raised in real time. After a project is released on an equity crowdfunding platform, the lead investor invests first. The proportion of money invested by a lead investor in the funding target could reflect his or her confidence in the project. This confidence may, in turn, influence other investors to follow. It has been argued that the entrepreneurs’ inclination to invest in their own projects represents a signal of project quality (Brealey et al., 1977). Entrepreneurs who are confident about their venture tend to hold more equity and sell less to the external investors (Vismara, 2016). Accordingly, the more the equity retained by entrepreneurs, the more positively is the signal perceived by external investors (Busenitz et al., 2005). This argument could also be applied to lead investors in the equity crowdfunding context. Lead investors are supposed to conduct due diligence on the projects they invest in. They have relatively more information on the projects than other potential investors before they make investment decisions. If they contribute a higher portion of the funding target after due diligence, this could be a signal that they believe the project has good quality and potential. In other words, the lead investors’ willingness to invest in projects signals the quality of the project.

Therefore, subsequent investors might perceive the projects receiving more investment from lead investors to have a higher probability of succeeding. It is obvious that investors tend to invest in projects that they believe are more likely to succeed because they do not want to waste their time and energy on unsuccessful projects. As a result, a higher percentage of investment by lead investors in the funding target might ultimately lead to a better fundraising performance. Based on this line of reasoning, we propose:

H1. The percentage of investment by lead investors in the funding target is positively related to the fundraising performance of equity crowdfunding.

In the financial industry, investment experience is vital (Hopp and Lukas, 2007) because investment experience of investors influences their returns (Elliott et al., 2008). Those who have more experience have a better chance of succeeding in future investments because they would have learned from their past experiences. The investing experience of lead investors could be successful or unsuccessful, both of which could facilitate future investment success. It is highly probable that past successful experience could predict continuous success in the future. Unsuccessful experiences could also be helpful for future investing success because investors can learn lessons from the unsuccessful experiences. Empirical evidence shows that investors with more investment experience are likely to be more successful in their subsequent investments. For example, Feng and Seasholes (2005) found that trading experience of individual investors positively affects disposition effect, which could bring about better investment performance. Similarly, Seru et al. (2009) demonstrated that investment performance improves as investors accumulate more investment experience.

In the context of equity crowdfunding, therefore, the experience of lead investors could send signals to other potential investors that the lead investors are more likely to succeed in the projects they invest in. In on Dajiatou, the lead investors display their investment experience on their profiles. Potential investors could perceive the projects invested in by experienced lead investors to be more likely to succeed, thus, choosing to support those projects. Compared with investors with no investing experience, those with experience are more likely to reassure potential investors, who might back the same project and facilitate the fundraising performance of equity crowdfunding. Based on this, we hypothesize:

H2. The investment experience of lead investors is positively related to the fundraising performance of equity crowdfunding.

The help provided by lead investors to the projects could drive the funding success of the projects. Young firms often face tremendous challenges. The help from external experts is important for their survival and development. It has been suggested that the help offered by investors could signal and certify the quality of a company (Lerner, 1994; Gompers, 1995). In the real world, venture capitalists and angel investors often offer help to the entrepreneurial firms, such as providing advice and networking opportunities, serving on boards of directors and advisors, and providing hands-on assistance and business intelligence (e.g., Hellmann and Puri, 2002; Madill et al., 2005). Like venture capitalists and angel investors, some lead investors in equity crowdfunding also claim to provide help to the projects. For example, in on Dajiatou, one of the lead investors claims, “I can help the firm to improve the business model, guide law-related practice, and facilitate cooperation with other firms.” This help is particularly vital for the survival and development of equity crowdfunding projects, which are usually very vulnerable at the early stages. Note that on equity crowdfunding platforms, most of the entrepreneurs are first-timers. Not surprisingly, they have limited entrepreneurial experience and executive abilities. Under this circumstance, the help offered by lead investors can help them conquer challenges and add values to their firms (Politis, 2008). Therefore, potential investors could perceive the help from lead investors to the projects as a signal of future success. As a result, the potential investors might choose to back those projects in which lead investors offer help, thus, driving the overall funding performance of equity crowdfunding. This reasoning leads to the following hypothesis:

H3. The help offered by lead investors to the project is positively related to the fundraising performance of equity crowdfunding.

## Materials and Methods

### Sample

Equity crowdfunding has grown rapidly in China with dozens of equity crowdfunding platforms currently. Some of these platforms have started to adopt the leader–follower investor mode, which provides the context for studying how lead investors influence follower investors’ decisions, which, in turn, affects fundraising performance. We collected the data from Dajiatou, which is among the top 10 equity crowdfunding platforms in China and can be used to test our hypotheses^2^. We collected data on equity crowdfunding projects listed on Dajiatou from 2012 to 2016. The information of each project will be listed on the webpage. We manually recorded the information that can be used to test our hypotheses. In total, we obtained a dataset of 215 projects, which include a broad range of industries, such as manufacturing, agriculture, and services.

### Measurement

#### Dependent Variable

The dependent variable is the fundraising performance of equity crowdfunding. We measure it with the ratio of pledge over funding goal following other studies (e.g., Zheng et al., 2014). Every equity crowdfunding project has a funding goal and duration at the beginning of its launch. The more money a project raises during the given duration, the better performance it has.

#### Independent Variables

The percentage of investment provided by lead investors is measured by the money invested by lead investors over the funding goal, which was set at the beginning of campaign. The investment experience of the lead investors is a binary variable. It is 1, if the lead investor has investment experience, else 0. The help offered by lead investors to a project is also a binary variable. It is 1, if the lead investor claims that he/she can offer help to the project, else 0.

#### Control Variables

We control for the crowdfunding goal and duration of the funding campaign because both variables could exert influence on the fundraising performance (Mollick, 2014; Zheng et al., 2014). The crowdfunding goal is measured by the total amount of money that an entrepreneur aims to raise for a particular project. Crowdfunding duration is the number of days from the start to the end of a project.

## Results

The descriptive statistics of the variables are shown in Table 1. The average funding goal of these projects is 1.2 million RMB. The average duration for a campaign is 75 days. Hierarchical regression analysis was used to test the hypothesized relationships (Bagozzi, 1994; Cohen et al., 2003). The regression results are presented in Table 2. In each step of the hierarchical regression analysis, the statistical significance of incremental R^2^ and F tests were evaluated. Model 1 includes two control variables, whereas Model 2 includes the control variables and independent variables. The variance inflation factor (VIF) for all the variables is less than 2, indicating that there is no serious colinearity (Hair et al., 1995).

**TABLE 1:** Descriptive statistics and variable correlations.

	**Mean**	**SD**	**1**	**2**	**3**	**4**	**5**
1. Funding goal	119.757	83.150	–	–	–	–	–
2. Duration	75.335	40.695	−0.357**	–	–	–	–
3. Percentage of lead investor funding in the target	0.132	0.101	−0.041	−0.051	–	–	–
4. Investment experience of lead investors	0.688	0.464	−0.046	−0.07 8	0.031	–	–
5. Help offered by lead investors	0.883	0.321	−0.033	−0.075	−0.206**	0.007	–
6. Performance	1.277	0.270	0.020	0.176**	0.306**	0.240**	0.001

**p < 0.01.

**TABLE 2:** Hierarchical multiple regression analysis.

**Variables**	**Model 1**			**Model 2**		
	**β**	***t***	**VIF**	**β**	***t***	**VIF**
Funding goal	0.095	1.314	1.146	0.146*	2.203	1.164
Duration	0.209**	2.905	1.146	0.272***	4.090	1.177
Percentage of lead investor funding in the target	–	–	–	0.338***	5.366	1.056
Investment experience of lead investors	–	–	–	0.257***	4.170	1.013
Help offered by lead investors	–	–	–	0.095	1.500	1.059
*F*	–	4.264*	–	–	11.515***	–
*R*^2^	–	0.039	–	–	0.197	–
AR^2^	–	0.039	–	–	0.177	–

Dependent variable: fundraising performance of equity crowdfunding project. *p < 0.05; **p < 0.01; ^∗∗∗^*p* < 0.001.

**FIGURE 2:**
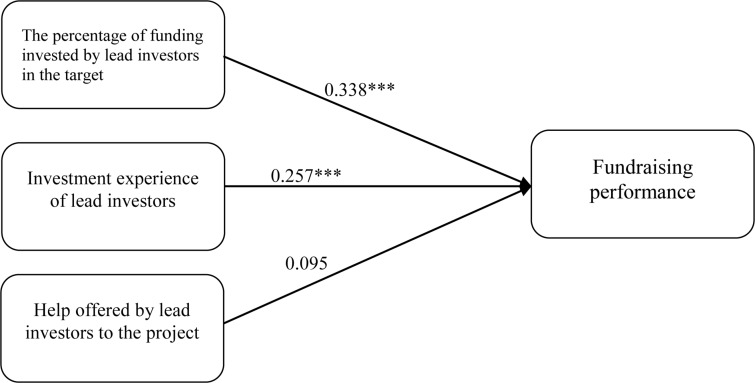
Results of the data analysis. *p < 0.05; **p < 0.01; ***p < 0.001.

Hypothesis 1 predicts that the percentage of money invested by lead investors in the funding target is positively related to the fundraising performance of equity crowdfunding. The results confirm this (*β* = 0.338, *p* < 0.001). Therefore, H1 is supported. Hypothesis 2 proposes that the investment experience of lead investors has a positive impact on fundraising performance. The results confirm this (*β* = 0.257, *p* < 0.001); thus, H2 is supported. Hypothesis 3 predicts that the help offered by lead investors to the entrepreneurial firm is positively related to fundraising performance. The results do not support this (*β* = 0.095, *p* > 0.1). Therefore, H3 is not supported. Figure 2 presents the results.

## Discussion

Psychological factors play a critical role in affecting investor decisions. We draw upon signaling theory and observational learning theory to examine how lead investors influence following investors psychologically, thus, affecting the overall fundraising performance in equity crowdfunding. We find that the percentage of money invested by lead investors in the crowdfunding goal and their investment experience are positively related to fundraising performance. We contribute to the literature on the factors of successful equity crowdfunding campaigns by introducing the role of lead investors. This is of great importance because the leader–follower model is nascent in equity crowdfunding in China. The knowledge of the influence of lead investors on the overall fundraising performance has important implications for entrepreneurs, lead investors, and equity crowdfunding platforms.

The help offered by lead investors was found to have no impact on fundraising performance. One possible explanation could be that the data did not allow us to capture the quality of help. As mentioned earlier, we treated help as a binary variable where it is 1 if lead investors offer help and 0 otherwise. However, the quality of the help could vary among lead investors and could affect fundraising performance. For example, those who can provide specific help needed by the projects may be more likely to attract following investors, while those who provide general help that does not fit well with the project may not be able to reassure other investors to follow. We believe that this limitation could provide a possible opportunity for future studies. Researchers can consider capturing the quality of lead investors’ help and investigate its influence on fundraising performance. In Table 2, the results show that the percentage of investment of lead investors has negative and significant relationship with help offered by lead investors. This finding is somewhat counterintuitive. This might be because lead investors who invest more to a project may have high confidence in that project. They tend to believe that project will be successful even without their help.

The findings of this study have theoretical and practical implications. Theoretically, there are four main implications as follows. First, to the best of our knowledge, we are the first to investigate the role of lead investors in the fundraising performance of equity crowdfunding. Given the emergence of lead investor–following investor model in the equity crowdfunding platforms in China, it is important to understand how lead investors impact the performance of equity crowdfunding. Agrawal et al. (2016) has introduced the syndicate model in the fundraising context. It also has lead investors in that model. However, the leader–follower model is different from the syndicate model and gains little attention in the literature. This paper has contributed the equity crowdfunding by unpacking the role of lead investors in the particular leader–follower model in the equity crowdfunding context. Second, we contribute to the signaling theory by highlighting the signaling role of lead investors in the context of equity crowdfunding. Signaling theory has been widely used in crowdfunding research (e.g., Carter and Manaster, 1990; Connelly et al., 2011; Migliorati and Vismara, 2014; Judge et al., 2015). However, the signaling role of lead investors has not been explored. This study is the first to investigate how lead investors signal the quality of projects and, thus, influence fundraising performance inequity crowdfunding. Third, we contribute to observational learning theory by providing supportive evidence from the context of equity crowdfunding. Our results suggest that following investors tend to make their decisions by watching the investment behaviors of lead investors. Fourth, we identified two factors, percentage of funding invested by lead investors and the investment experience of lead investors, as the predictive variables on the equity crowdfunding performance. These findings enriched the previous literature that attempted to unpack the antecedents of equity crowdfunding success (Ahlers et al., 2015; Lukkarinen et al., 2016; Vismara, 2016).

Practically, our study has three contributions. First, the findings indicate that lead investors are important people that influence the performance of equity crowdfunding. In particular, the proportion of money invested by lead investors and their investment experience positively influences fundraising performance in equity crowdfunding. These findings indicate that entrepreneurs should consider soliciting lead investors to invest more on their projects and encourage them to explicitly display their investment experience on the websites, thus, attracting more following investors. Second, the equity crowdfunding platforms should screen lead investors by their capital and investment experience to improve fundraising performance of the projects. Specifically, equity crowdfunding platforms should consider taking steps to attract and retain lead investors who are willing to invest more money on projects and who have investment experience. Third, the findings highlight the importance of lead investors in the fundraising performance of equity crowdfunding. Lead investors, themselves, should be responsible for every project they take the lead to invest because their behaviors will influence the success of the project and other’s benefits.

## Limitations and Future Research Direction

There are several limitations in this study. First, we only focus on the Chinese context. The findings may not hold in other countries and cultures. Culture may play a role in affecting the results. For example, people in a different level of uncertainty avoidance may have a different tendency to follow others when making decisions. Hence, the results should be generalized with caution. Future studies could replicate the current study in other contexts to see if the results are consistent. Second, the data in this study only includes 215 projects from one platform. Future studies could collect larger datasets across more platforms to get more statistically salient results. Third, the secondary data from the crowdfunding platforms prevented us from measuring some variables in detail. For example, we did not capture the quality of help by lead investors. Likewise, we only use binary way to measure investment experience, not Likert scale, which might be better to measure the level of their experience. Future studies could consider combining secondary and subjective data to better measure the variables.

Scholars could also consider other types of variables associated with lead investors. We only focused on the amount of money invested by lead investors, their investment experience, and the help offered by them. The influence of other variables, such as education background and social capital of lead investors can also be explored. With regard to the control variables, we follow previous research to include crowdfunding duration and funding goal. Future research could consider other control variables into the model.

## Conclusion

Despite increasing attention on the factors of fundraising performance inequity crowdfunding, little is known about the role of lead investors in this. This study explores how lead investors influence fundraising performance inequity crowdfunding by applying signaling theory and observational learning theory. Drawing on a sample from a Chinese equity crowdfunding platform, we find that the proportion of funding invested by lead investors in the funding target and their investment experience are positively related to fundraising performance. This study is the first to consider lead investors as an important party when exploring factors that influence fundraising performance inequity crowdfunding. We believe that this study is a substantial contribution to the literature about the antecedents of funding performance inequity crowdfunding. It also has practical implications for entrepreneurs and equity crowdfunding platforms.

## Data Availability Statement

The datasets generated for this study are available on request to the corresponding author.

## Author Contributions

TS designed the overall research, collected data, and analyzed data. JM wrote part of the manuscript. BZ conducted literature review. WH helped the first author to analyze data. FF helped the first author to collect data.

## Conflict of Interest

The authors declare that the research was conducted in the absence of any commercial or financial relationships that could be construed as a potential conflict of interest.

## Footnotes

1 Source: https://dazeinfo.com/2016/01/12/crowdfunding-industry-34-4-billion-surpass-vc-2016/

2 Apart from Dajiatou, other equity crowdfunding platforms in China also adopt the leader–follower model. However, only Dajiatou displays the lead investors’ complete information correctly. Hence, we chose this website as our sample source.
